# Akt/mTOR Pathway Agonist SC79 Inhibits Autophagy and Apoptosis of Oligodendrocyte Precursor Cells Associated with Neonatal White Matter Dysplasia

**DOI:** 10.1007/s11064-023-04057-w

**Published:** 2023-11-28

**Authors:** Zhongni Li, Feng Zhang, Li Huang, Jiehong Deng, Yutong Pan, Ting Xu, Jingyi Liu, Na Gao, Rongrong Duan, Chunyan Shao, Chan Wu, Minrong Wang, Liqun Lu

**Affiliations:** 1 Department of Pediatrics, The First Affiliated Hospital of Chengdu Medical College, No. 278, Middle Section of Baoguang Avenue, Xindu District, Chengdu, 610500 Sichuan Province People’s Republic of China; 2 Clinic Medical College, Chengdu Medical College, No. 783 Xindu Avenue, Xindu District, Chengdu, 610500 Sichuan Province People’s Republic of China

**Keywords:** SC79, Oligodendrocyte precursor cells, White matter dysplasia, Apoptosis, Autophagy

## Abstract

White matter dysplasia (WMD) in preterm infants due to intrauterine inflammation is caused by excessive apoptosis of oligodendrocyte precursor cells (OPCs). In recent years, studies have found that excessive autophagy and apoptosis are highly interconnected and important in infection and inflammatory diseases in general. Therefore, in this study, we aimed to confirm whether regulation of autophagy by using the Akt phosphorylation agonist SC79 can inhibit abnormal apoptosis of OPCs and promote myelin maturation and white matter development in neonatal rats with WMD. We investigated the effect of inflammation on oligodendrocyte development in P0 neonatal rats by intracerebellar injection of LPS, and collected brain tissue at P2 and P5. Immunohistochemical and immunofluorescence staining were used to evaluate white matter damage, while immunofluorescence staining, terminal deoxynucleotidyl transferase dUTP nick end labeling analysis (TUNEL), and western blotting were used to evaluate autophagy and apoptosis. First, we observed that white matter development was arrested and white matter fiber maturation was impaired in LPS-inflicted pups compared with those in the sham-operated group. Second, treatment with SC79 reduced the levels of LC3II, caspase 3, caspase 9, and Bax/Bcl-2 and increased the levels of p62, p-Akt, and p-mTOR in the brain tissue of neonatal rats. Finally, SC79 treatment inhibited OPC apoptosis by increasing the binding of Beclin 1 to Bcl-2, which promoted OPC differentiation and maturation. However, the opposite results were observed after rapamycin administration. Taken together, our results suggest that SC79 can inhibit the abnormal apoptosis of OPCs caused by excessive autophagy through the Akt/mTOR pathway and that SC79 is a potential therapeutic agent for WMD in preterm infants.

## Introduction

White matter dysplasia (WMD) comprises a variety of diseases characterized by developmental abnormalities of CNS white matter. WMD usually exists after white matter injury (WMI) caused by intrauterine inflammation. WMI is the main form of brain injury in preterm infants [1, 2]. Approximately 50–80% of preterm and very preterm infants have cognitive and behavioral deficits, learning disabilities, and vision and hearing problems, which may be related to WMI. However, no targeted treatment for reducing or preventing its occurrence exists [3, 4]. Among the probable reasons behind WMI, intrauterine inflammation during pregnancy is a common risk factor for preterm birth and white matter damage. The inflammatory cascade at the maternal-fetal interface penetrates the fetal blood-brain barrier and leads to white matter damage [5]. This leads to impaired white matter development, which caused WMD. Perinatal infection or inflammation is closely related to WMI in preterm infants [6]. Intrauterine endotoxin exposure can lead to continuous activation of microglia in the neonatal periventricular white matter [7]. Numerous proinflammatory cytokines are released from activated microglia and induce apoptosis of oligodendrocyte precursor cells (OPCs), thereby impairing the proliferation, differentiation, migration, and maturation of oligodendrocyte lineage cells in the white matter [8]. Promoting WMD recovery is an method to rescue white matter damage. Administration of activated protein C, which has anti-inflammatory and anti-apoptotic activities, in neonatal rats with WMI induced by intrauterine infection significantly reduces the production of lipopolysaccharide (LPS)-induced proinflammatory factors such as tumor necrosis factor-α, interleukin-6, and interleukin-1β and reduces the degree of apoptosis of periventricular white matter cells and brain edema. Furthermore, hypomyelination and white matter structural disorders in the periventricular region significantly improve [9]. Therefore, inhibiting the inflammatory response or abnormal apoptosis of OPCs may contribute to the repair of the inflammatory myelin sheath after injury.

Inflammation leads to excessive apoptosis of OPCs; however, the underlying mechanism remains unclear. When cells are exposed to inflammatory stimuli, excessive activation of autophagy may induce abnormal apoptosis [10]. Inhibiting the excessive activation of mitochondria-mediated apoptosis and autophagy protected mice from LPS-induced acute lung injury [11]. Furthermore, melatonin, a hormone with broad disease-protective effects, inhibited autophagy by decreasing the expression of the autophagy proteins Beclin 1 and LC3-phosphatidylethanolamine conjugate (LC3-II) and attenuated LPS-induced proinflammatory cytokine responses in human meibomian gland epithelial cells in a cell model of LPS-induced meibomian gland dysfunction. Meanwhile, the level of cleaved-caspase 3 and ratio of Bcl-2 associated X apoptosis regulator (Bax)/Bcl-2 ratio decreased synchronously, which reduced the apoptosis of human meibomian gland epithelial cells [12]. However, in some animal models of WMI, increased autophagy levels coexist with abnormal apoptosis of OPCs [13]. In a rat model of cystic periventricular leukomalacia, strong morphological characteristics of enhanced autophagy were observed at the ultrastructural level. Injection of the autophagy inhibitor 3-methyladenine not only prevented the induction of autophagy but also the activation of the apoptotic protein caspase 3 and caused the reduction of subcortical white matter thickness after injury. The coexistence of neuronal apoptosis and autophagy has also been observed in a rat model of hypoxia–ischemia-induced periventricular leukomalacia [14]. Some studies have suggested that abnormal apoptosis induced by excessive autophagy may be related to the abnormal binding state of the anti-apoptotic protein Bcl-2 and autophagy initiation protein Beclin 1 in cells. Under normal conditions, the binding state of Bcl-2 and Beclin 1 inhibits autophagy; however, under inflammatory stimulation and cell stress, it leads to the destruction of the Beclin 1/Bcl-2 complex and dissociation of Bcl-2, which is conducive to the initiation of apoptosis by pro-apoptotic proteins and the transition from autophagy to apoptosis [15]. These findings were confirmed using a cellular model of chronic cerebral hypoperfusion. The interaction of Bcl-2 and Beclin 1 increased after psyllium husk treatment, while free Beclin 1 and LC3-II levels decreased, thereby inhibiting the excessive autophagy of cells and abnormal apoptosis of OPCs [16].

Mechanistic target of rapamycin kinase (mTOR), a downstream effector of the serine/threonine kinase Akt, negatively regulates autophagy and is associated with various downstream signaling pathways, including apoptosis and inflammatory responses [17, 18]. Akt directly phosphorylates and activates mTOR. As discovered in an in vitro study on cerebral ischemia-reperfusion injury, following cerebral ischemia-reperfusion injury, complement activation produces C5a, which interacts with C5a receptor 1 to inactivate mTOR, leading to autophagy activation through the Akt/mTOR pathway and eventually neuronal apoptosis and brain damage [19]. This finding suggests that the Akt/mTOR pathway is involved in regulating autophagy and apoptosis.

Drugs that activate the Akt/mTOR pathway are effective treatment modalities for various diseases. Plant-derived compound gastrodia-20 C played an anti-neuroinflammatory role and could regulate autophagy in a mouse microglioma cell line activated by LPS through the Akt/mTOR signaling pathway [17]. In a cell model of LPS-induced necrotizing enterocolitis, β-carotene could attenuate autophagy and apoptosis of intestinal epithelial cells by activating the Akt/mTOR signaling pathway [20].

We hypothesized that neonatal WMD caused by intrauterine inflammation during pregnancy may be due to excessive apoptosis of OPCs. Whether excessive autophagy of OPCs can lead to increased apoptosis and excessive autophagy can regulate OPCs apoptosis through the Akt/mTOR pathway remains unclear. To address this issue, we investigated the role of the Akt phosphorylation agonist SC79 in neuroinflammation simulated by LPS in neonatal Sprague–Dawley (SD) rats.

## Methods

### Rats

The animals required for this study were purchased from Chengdu Dashuo Experimental Animal Co., LTD. (Sichuan, China), and all were 3–4 months old, including female SD rats weighing 300–350 g and male SD rats weighing 350–400 g. This experiment complied with the UK Animal Science Procedure guidelines and was performed in accordance with the UK Animals (Scientific Procedures) Act of 1986 and the National Research Council Guide for the Care and Use of Laboratory Animals. This study was also approved by the Animal Ethics Committee of Chengdu Medical College. This study has not been registered.

Male and female rats were randomized in a 2:1 ratio, allowed to eat and drink ad libitum, and acclimated in standard cages maintained at 22 ± 2 °C, a relative humidity of 50–60%, and a 12 h light–dark cycle for 1 week. As previous literature reported that sex has little effect our experiment, we did not distinguish between the sexes of the pups. Necessary precautions were taken to minimize pain and stress.

We included term male and female SD rat pups born between E21 and E22. To ensure the comparability of the two groups, we limited the number of pups to 11–13 pups per group. A total of 120 rats were included, of which 42 were assigned to the sham-operation group and 78 were assigned to the WMD model group. P0, the first time point after birth, is defined as no more than 6 h after birth. At P1–P5 (between days 1 and 5 after birth), rodent brains are equivalent to human brains at 23–32 weeks gestation. This time frame is therefore suitable for modeling brain injury in preterm infants [21]. Neonatal rat body weight was monitored at P0, P2, and P5, and brain tissues were randomly collected at different time points after birth (P2 and P5). Animals that died were excluded from the study. Adult rats continued to be fed or were used for other studies following the “3R” (replace, reduce, and refine) principle [22]. In this study, each group included six samples, and three independent replicates were performed for each sample.

### Intracerebroventricular Injection

- After stabilization of the intrauterine inflammation group in the normal environment for 6 h, 1 µL LPS (L2880-10 mg; Sigma, St. Louis, Missouri, USA) dissolved in 0.1 mg/mL saline (total dose 1 mg/kg) was stereotaxically injected into the ipsilateral hemisphere ventricle of rat pups (coordinates: 2 mm posterior, 1.5 mm transverse, 3 mm below the skull surface) at a flow rate of 0.5 µL/min [23, 24]. The LPS dose was determined from a concentration gradient established in a preliminary study. The body weight of neonatal rat was fixed at 6.0 ± 1.5 g, and the total dose of LPS was 6.0 ± 1.5 µg. After LPS injection, rats in the intervention group were injected with 1 µL SC79 (S7863, 20 µmol/L dissolved in dimethyl sulfoxide, diluted in normal saline according to the manufacturer’s instructions) or rapamycin (RAPA; S1039, 5 mM dissolved in dimethyl sulfoxide, diluted in normal saline according to the manufacturer’s instructions) at P2 in the lateral ventricle. Sham-operated rats were injected with the same volume of solvent. Brain tissue was sampled from the cerebral hemisphere contralateral to the ventricular injection site. To reduce pain, rats were deeply anesthetized with an intraperitoneal injection of 4% pentobarbital sodium (200 mg/kg body weight) prior to euthanasia. After surgery, all neonatal rats were numbered according to a random number table generated in Excel (Microsoft, Redmond, WA, USA). Random numbers were sorted from small to large and assigned to different groups. Numerical sample identifiers were used during experimental procedures and data analysis, and the researchers were blinded to the treatment.

### Histopathology

Rat brain tissues were collected, embedded in paraffin, and sectioned for pathological examination. Briefly, animals were deeply anesthetized by intraperitoneal injection of 4% sodium pentobarbital (200 mg/kg body weight) and perfused with normal saline via the cardiac vein, followed by injection of 4% paraformaldehyde. Tissues were fixed in paraformaldehyde at 22 ± 2 °C for 72 h, dehydrated in gradient alcohol, embedded in paraffin, and sectioned in the coronal plane (5 μm thick). Tissues from the corpus callosum to the dorsal end of the hippocampus were stained with hematoxylin and eosin (H&E; Cat# G1121; Solarbio, Beijing, China). Randomly selected microscope fields were captured from six serial sections from each brain using a Leica microscope (DM4000B; Leica, Wetzlar, Germany) and 40x objective magnification and analyzed by a blinded investigator using ImageJ 1.8.0 (RRID: SCR_003070; National Institutes of Health [NIH], Bethesda, MD, USA).

### Immunohistochemistry and Immunofluorescence

Myelin basic protein (MBP) and 2ʹ,3ʹ-cyclic nucleotide 3ʹ-phosphodiesterase (CNPase) expression levels were quantified by H&E, immunofluorescent, and immunohistochemical staining to determine oligodendrocyte maturity and evaluate the degree of WMD. For immunofluorescence and immunohistochemical analyses, tissues were blocked with 1× phosphate-buffered saline, 10% normal goat serum, and 0.1% Triton X-100 for 10 min. For immunohistochemical analysis, 3% hydrogen peroxide was added for 10 min to inhibit endogenous peroxidase activity. The sections were then incubated with a primary antibody solution containing goat anti-MBP (1:100; RRID: AB_305869; Abcam, Cambridge, UK), goat anti-CNPase (1:100; RRID: AB_2082593 Abcam), anti-Beclin 1 (1:100, Cat#66665-1-Ig, Proteintech, China), anti-Bcl-2 (1:100, Proteintech, RRID: AB_2227948), and OPC-specific O4 (1:100; Cat#MAB1326-SP; R&D Systems, Minneapolis, MN, USA) antibodies. Tissues were subsequently incubated with enzyme-conjugated goat anti-mouse immunoglobulin G (IgG) secondary antibody (Cat #SP-900; ZSGB-Bio, Beijing, China) for 15 min at 37 °C, followed by incubation with 3,3ʹ-diaminobenzidine working solution (Cat #ZLI-9019; ZSGB-Bio). MBP signal intensity was expressed as mean MBP fluorescence intensity. ImageJ 1.8.0 was used to determine the optical density of each image pixel. The integrated optical density (IOD) was measured, and the average optical density (AOD; IOD/target distribution area) was calculated. To evaluate the binding of Beclin 1 to Bcl-2 in O4+ cells, the antibodies for each were simultaneously used for labeling and observed under a laser confocal microscope (Nikon A1, ver.4.10).

For immunofluorescent staining, the sections were treated with an appropriate fluorescein isothiocyanate-conjugated secondary antibody (1:300; Cat #550,066; ZenBio, China).

### Terminal Deoxynucleotidyl Transferase dUTP Nick End Labeling Assay

Paraffin-embedded sections were deparaffinized, rehydrated, and antigen-repaired in citrate buffer (pH 6.0) for 8 min and then incubated with 20 mg/mL proteinase K (Cat #ST533; Beyotime, Shanghai, China) dissolved in Tris/HCl for 30 min at 22 ± 2 °C. The sections were subsequently incubated in a terminal deoxynucleotidyl transferase (TdT) enzyme reaction solution (Cat# KGA700; KeyGen Biotech, Nanjing, China) for 1 h at 37 °C in a wet box. O4-antibody (1:100; Cat #MAB1326-SP; R&D Systems, Minneapolis, MN, USA) and terminal deoxynucleotidyl transferase dUTP nick end labeling (TUNEL; Cat #G1501-100T; Servicebio, Wuhan, China) fluorescent double staining was used at P2 to determine whether SC79 can reduce excessive OPC apoptosis.

### Western Blotting

Western blotting was performed using a dioctanoic acid protein detection kit (Cat #PC0020; Solarbio). Samples (40 to 60 µg/10 µL) were separated using 8% or 12% sodium dodecyl sulfate-polyacrylamide gel electrophoresis. Polyvinylidene difluoride membranes were subsequently used for protein transfer, and samples were incubated overnight at 4 °C with the following primary antibodies: anti-p-Akt (Ser473) (1:2000; RRID: AB_2315049; Cell Signaling Technology), anti-Akt (1:1000; RRID: AB_915783; Cell Signaling Technology), anti-p-mTOR (1:2000; Cat #R25033; ZenBio), anti-mTOR (1:2000; Cat #380,411; ZenBio), anti-p62 (1:2000; RRID: AB_10694431; Proteintech), anti-LC3 (1:1000; RRID: AB_2137737; Proteintech), anti-Bax (1:10 000; RRID: AB_2061561; Proteintech, Planegg-Martinsried Germany), anti-Bcl-2 (1:1000, Proteintech, RRID: AB_2227948), anti-cleaved caspase 3 (1:1000; Cat #341,034; ZenBio), anti-caspase 3 (1:1000; RRID: AB_331439; Cell Signaling Technology), anti-caspase 9 (1:500; RRID: AB_2068632; Proteintech), and anti-β-actin (1:5000; Cat #CL594-66009; Proteintech). Goat anti-mouse or anti-rabbit IgG secondary antibodies were incubated with horseradish peroxide (1:500; RRID: AB_449890; Abcam, Cambridge, UK). Signals were detected using a bioimaging system (ChemiDoc XRS+; Bio-Rad), and ImageJ software was used to analyze the band intensity.

### Statistical Analysis

Data are presented as the mean ± standard error of the mean (SEM). Outlier testing was not performed due to the small sample size. The Shapiro–Wilk test was used to evaluate normality of the experimental data. An unpaired Student’s *t* test was used to compare the means of two groups, and one-way or two-way analysis of variance (ANOVA) was used to compare multiple groups. Prism 8.02 software (RRID: SCR_002798; GraphPad, San Diego, California, USA) was used for the statistical analyses. P < 0.05 was considered statistically significant.

## Results

### White Matter Maturation Disorders in Neonatal Rats After Intracerebroventricular Injection of LPS

**Fig. 1:**
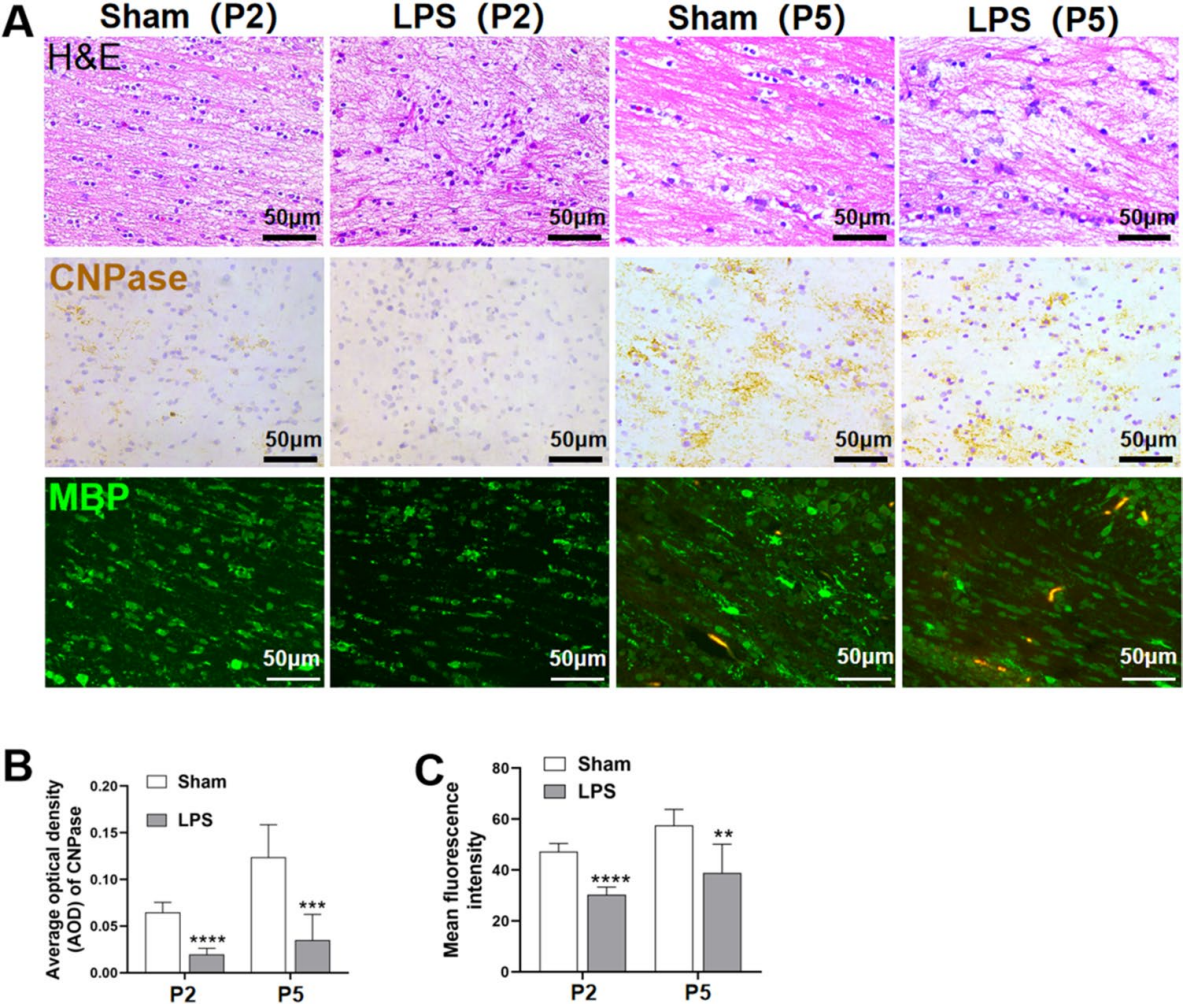
White matter maturation disorders in neonatal rats after intracerebroventricular injection of LPS. **a** White matter of rats in the sham and LPS groups stained with H&E at P2 and P5. Scale bar = 50 μm. **a**, **b** Immunohistochemical analysis of CNPase expression at P2 and P5 and the AOD (∗∗∗∗P < 0.0001 at P2, ∗∗∗P < 0.001 at P5; n = 6). **a**, **c** Mean signal intensity of MBP (∗∗∗∗P < 0.0001 at P2, ∗∗P < 0.01 at P5; n = 6). Data are represented as mean ± SEM. Scale bar = 50 μm

Brain tissue was H&E stained (Fig. 1a) to compare changes in nerve fibers in the periventricular white matter of the sham and LPS groups at time points P2 and P5, which represent critical periods of white matter development. In the LPS group, cavitation around white matter cells increased after P2. Specifically, the white matter was lightly stained with a sparse structure and cribriform changes. At P5, the LPS group exhibited disordered white matter arrangement with glial scar formation, whereas the sham-operated group exhibited closely arranged, mature white matter. These pathological changes confirmed successful establishment of a LPS model caused by intrauterine inflammation in neonatal rats. Immunostaining for CNPase and MBP in six randomly selected periventricular white matter regions (Fig. 1a–c) revealed significantly decreased CNPase AOD in the cortical white matter of the LPS group compared with that in the sham-operated group at P2 (P < 0.0001) and P5 (P < 0.001) (Fig. 1a, b). The mean fluorescence intensity of MBP was reduced in the LPS group compared with that in the sham-operated group at P2 (P < 0.0001) and P5 (P < 0.01), and MBP + cells were sparsely arranged in the LPS group (Fig. 1a, c).

### Effect of LPS Treatment on Apoptosis and Apoptotic Protein Expression in OPCs

**Fig. 2:**
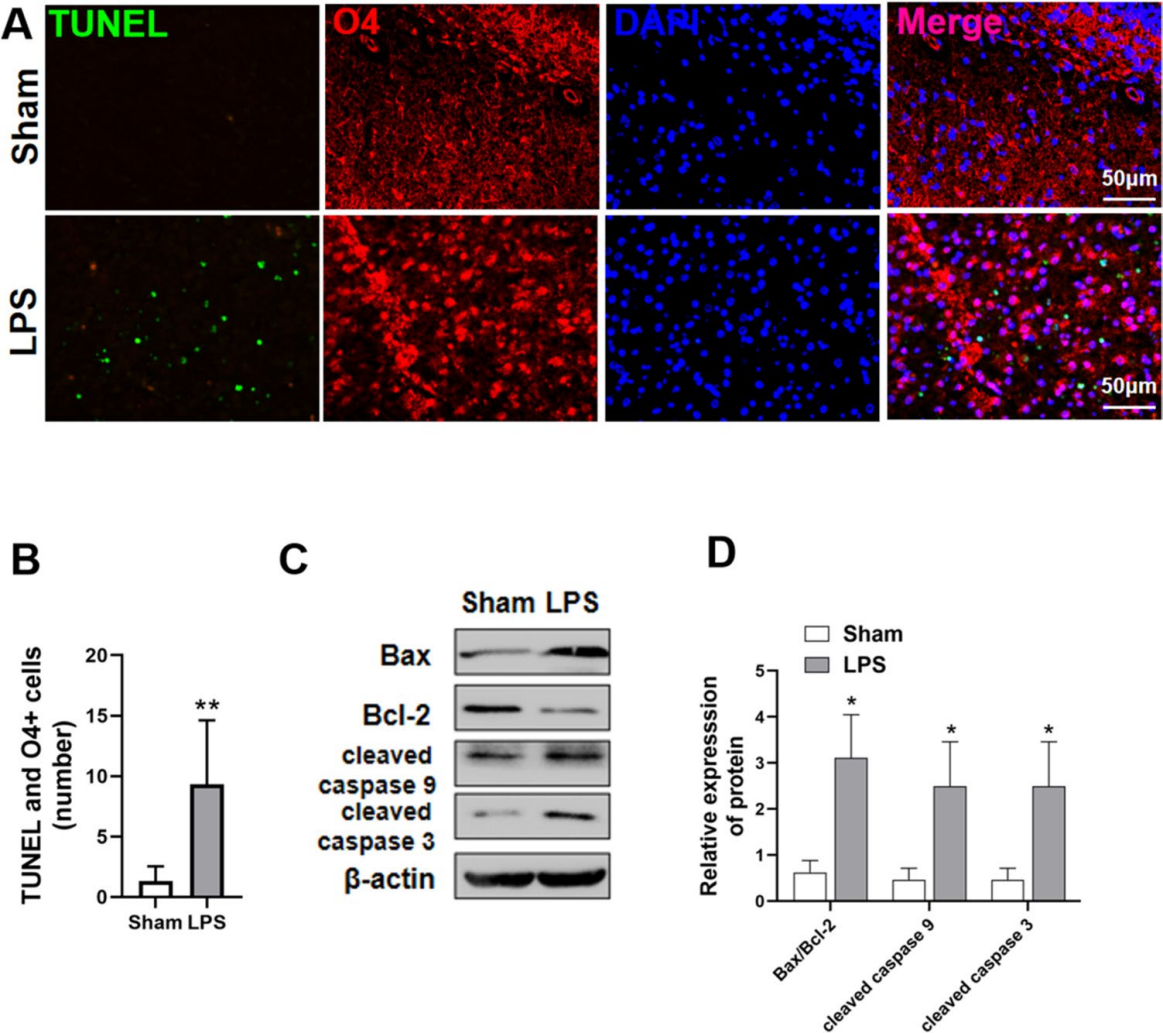
LPS increased OPC apoptosis and up-regulated apoptotic protein expression. **a**, **b** TUNEL staining of tissues from sham-operated and LPS-treated groups. TUNEL-positive cells are indicated in green. O4+ cells are indicated in red. (n = 6; ∗∗P < 0.01, P2). **c**, **d** Western blotting indicating relative expression of apoptosis-related proteins (Compared with the grey value of β-actin ), including Bax/Bcl-2, cleaved caspase-3, and cleaved caspase-9, in the brain tissue of rats in the sham-operated and LPS groups (n = 6; ∗P < 0.05). Data are represented as mean ± SEM. Scale bar = 50 μm

The number of O4+ apoptotic cells was significantly higher in the LPS group rats than in the sham-operated group rats (P < 0.01) (Fig. 2a, b). LPS-treated rats exhibited increased Bax/Bcl-2 (P < 0.05), cleaved caspase-3 (P < 0.05), and cleaved caspase-9 (P < 0.05) expression and activation compared with their levels in sham-operated rats (Fig. 2c, d).

### Autophagy Protein Expression and Activation in the Cortical Brain Tissue of Neonatal Rats

**Fig. 3:**
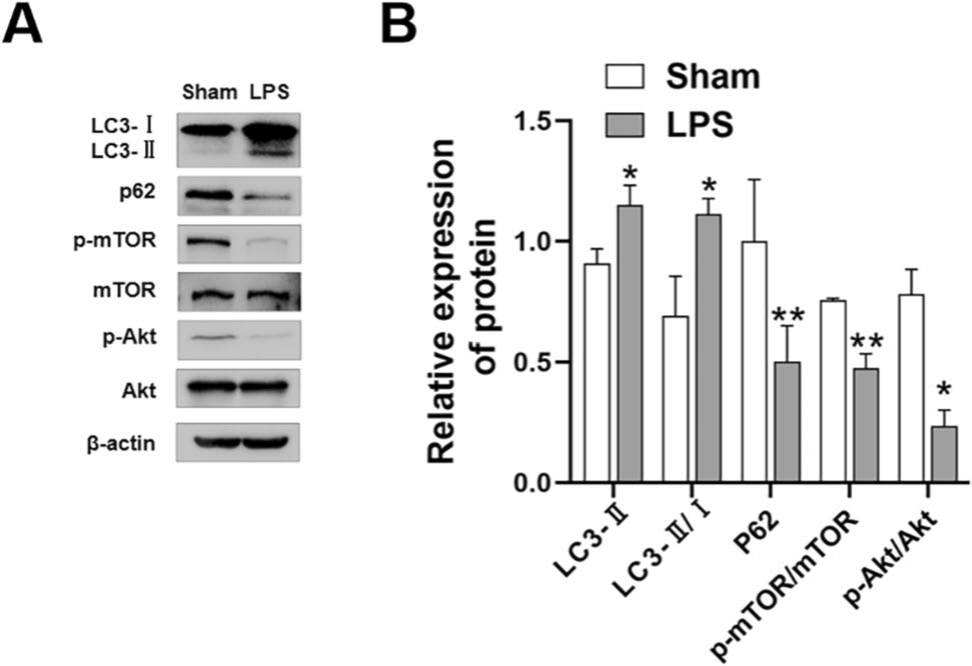
Autophagy protein expression and activation in the brain tissue of neonatal rats at P2. **a**, **b** Relative expression of autophagy-related proteins (Compared with the grey value of β-actin ) in the brain tissue of neonatal rats in the sham-operated and LPS groups evaluated with western blotting. LC3-II (∗P < 0.05), LC3-II/I (∗P < 0.05), p62 (∗∗P < 0.01), p-Akt/Akt (∗P < 0.05), p-mTOR/mTOR (∗∗P < 0.01). n = 6; data are presented as mean ± SEM

In the LPS group rats, LPS treatment induced activation of the autophagy marker protein LC3, thereby increasing LC3-II expression (P < 0.05) and the LC3-II/LC3-I ratio (P < 0.05). (Fig. 3a, b). Simultaneously, expression of the autophagy flux protein p62 was significantly lower than that in the sham-operated group rats (P < 0.01). Furthermore, Akt and mTOR phosphorylation levels were significantly lower than those in the sham-operated group rats (p-Akt/Akt, P < 0.05; p-mTOR/mTOR, P < 0.01).

### Autophagy Initiation Protein Beclin 1 and Anti-apoptotic Protein Bcl-2 Complexation

**Fig. 4:**
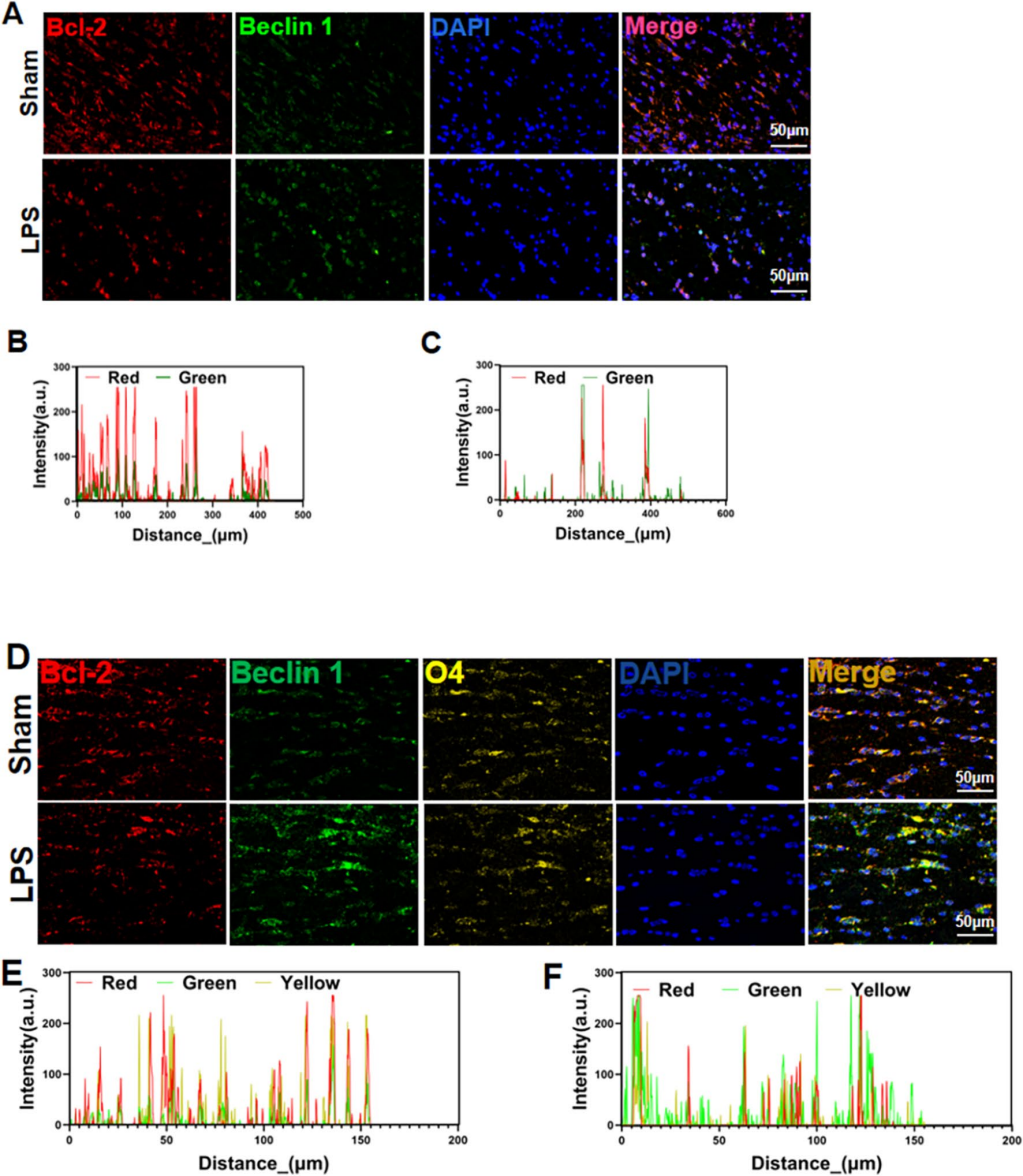
Autophagy activation in white matter. **a**–**c** Fluorescent staining and colocalization analysis of anti-apoptotic protein Bcl-2 (red) and autophagy initiation protein Beclin 1 (green) in the periventricular white matter of neonatal rats. **d**–**f** Confocal microscopy and colocalization analysis of Bcl-2 (red), Beclin 1 (green), and OPC marker O4 (yellow) in the periventricular white matter of neonatal rats. n = 6; data are presented as mean ± SEM. Scale bar = 50 μm

Fluorescence microscopy revealed increased Beclin 1 expression, decreased Bcl-2 expression, and decreased Beclin 1/Bcl-2 complexation in the white matter of the LPS group rats (Fig. 4a–c). Confocal microscopy revealed decreased Beclin 1/Bcl-2 complexation in O4+-labeled OPCs (Fig. 4d–f).

### Effect of SC79 Treatment on White Matter Damage and Myelin Development and Maturation

**Fig. 5:**
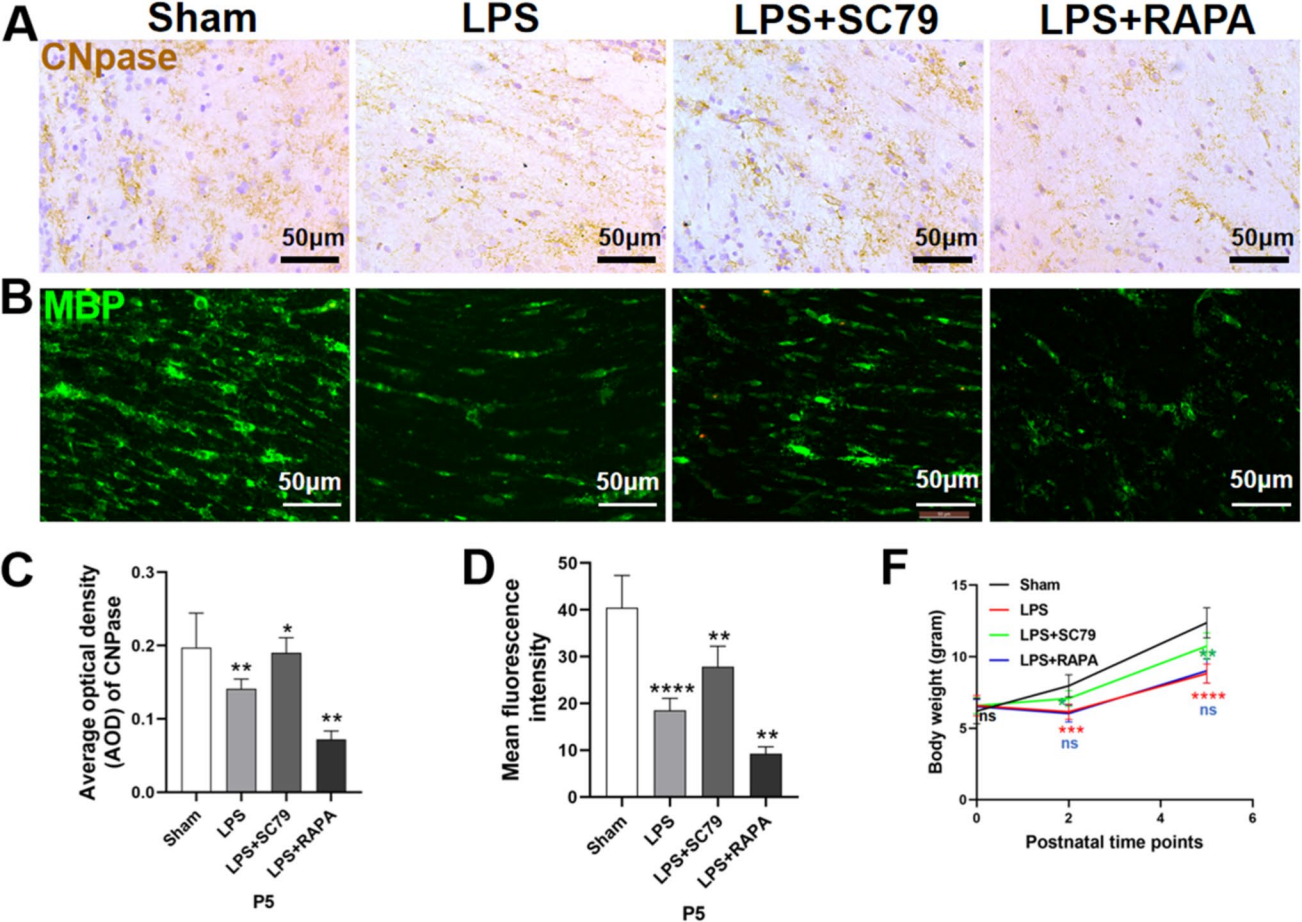
Effect of SC79 treatment on OPC maturation and myelination. **a**, **c** Immunohistochemical analysis and AOD of CNPase expression in the periventricular white matter of neonatal rats at P5 (n = 6; ∗P < 0.05, ∗∗P < 0.01). **b**, **d** Immunofluorescence and mean fluorescence intensity of MBP in the periventricular white matter of neonatal rats at P5 (n = 6; ∗∗P < 0.01, ∗∗∗∗P < 0.0001). Data are presented as mean ± SEM. Scale bar = 50 μm. **f** Neonatal rat body weight at the postnatal time points P0, P2, and P5 (n = 8)

CNPase immunolabeling (Fig. 5a, c) and MBP immunofluorescent labeling (Fig. 5b, d) were used to examine white matter formation in the midbrain of rats at P5. LPS rats treated with SC79 exhibited higher CNPase (P < 0.05) and MBP (P < 0.01) levels, as well as more ordered, regular myelin sheath arrangement and better white matter development than those in the rats of the LPS group. RAPA-treated rats exhibited decreased CNPase (P < 0.01) and MBP (P < 0.01). LPS-treated neonatal rats had significantly lower body weights at P2 (P < 0.001) and P5 (P < 0.0001) and slower growth rates (Fig. 5f) than those in the rats of the sham-operated group. SC79-treated pups gained more weight at both P2 (P < 0.05) and P5 (P < 0.01) than LPS-injected newborn rats.

### Effect of SC79 Treatment on Akt and mTOR Phosphorylation and Autophagy-Related Protein Expression

**Fig. 6:**
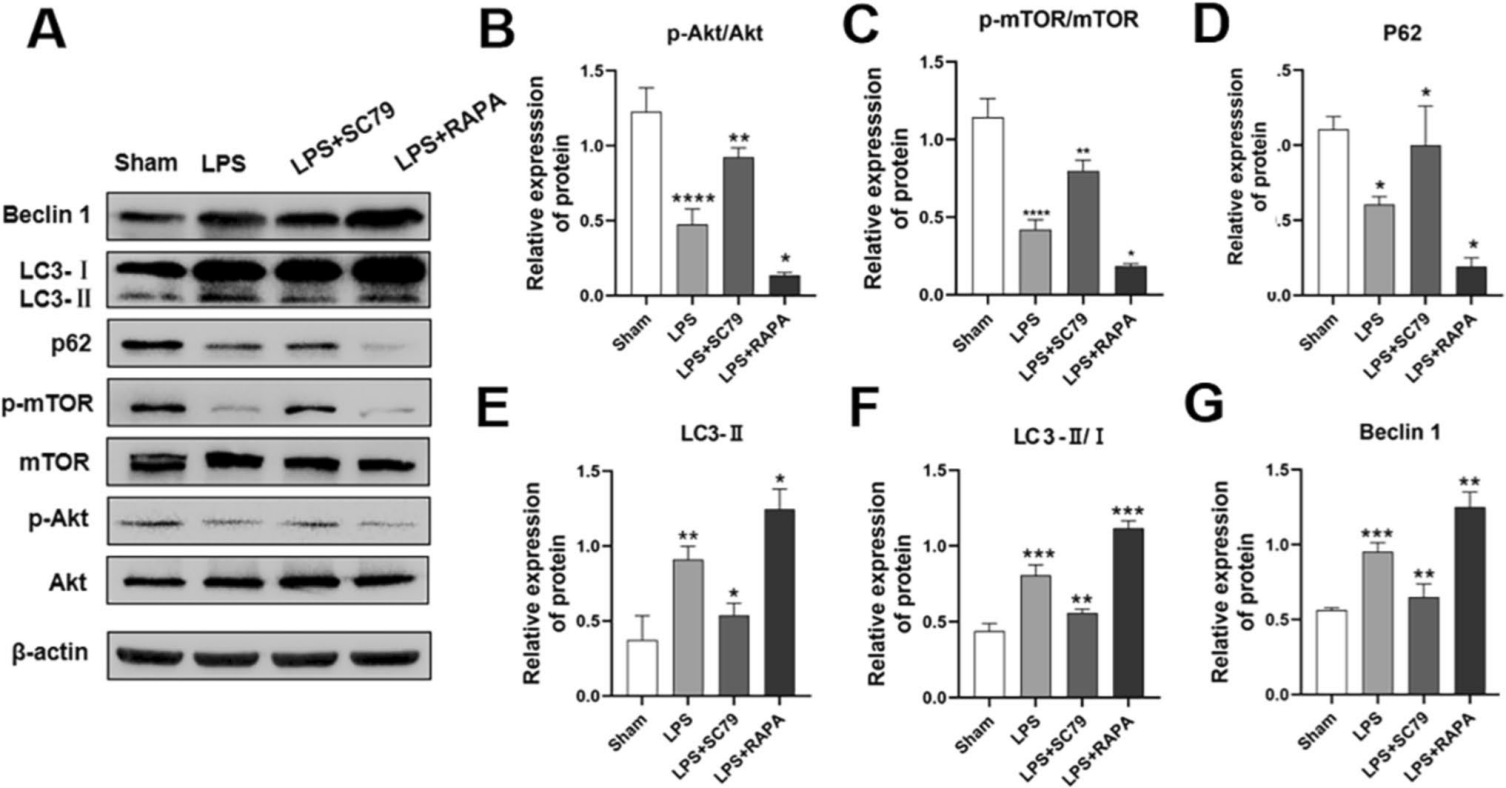
Effect of SC79 treatment on the Akt/mTOR signaling pathway. **a**–**g** Western blotting was used to quantify Beclin 1, LC3-II, LC3-II/I, p62, p-Akt/Akt, and p-mTOR/mTOR expression in the brain tissue of neonatal rats in the Sham, LPS, LPS+SC79, and LPS+RAPA groups at P2 (Compared with the grey value of β-actin ) (∗P < 0.05; ∗∗P < 0.01, ∗∗∗P < 0.001, ∗∗∗∗P < 0.0001). n = 6; data are presented as mean ± SEM

Western blot analysis (Fig. 6) revealed decreased p-Akt/Akt (P < 0.0001) and p-mTOR/mTOR (P < 0.0001) in the cortical brain of neonatal rats in the LPS group compared with that in the sham-operated group. Akt and mTOR phosphorylation increased significantly in the SC79-treated group (p-Akt/Akt, P < 0.01; P-mTOR/mTOR, P < 0.01), whereas RAPA treatment decreased p-Akt/Akt (P < 0.05) and p-mTOR/mTOR (P < 0.05). Compared with the sham-operated group rats, the LPS group rats exhibited significantly increased LC3-II (P < 0.01), LC3-II/I (P < 0.001) and significantly reduced p62 expression (P < 0.05). SC79 treatment significantly reduced the LC3-II ratio (P < 0.05), LC3-II/I (P < 0.01) whereas RAPA treatment significantly increased the LC3-II (P < 0.05), LC3-II/I (P < 0.001) and significantly decreased p62 expression (P < 0.05). The expression of Beclin 1 increased in the brain tissue of the pups after LPS injection (P < 0.001), and this increase was suppressed after the administration of SC79 (P < 0.01). Beclin 1 expression was increased after RAPA injection (P < 0.01).

### Effect of SC79 Treatment on Apoptotic Protein Expression and Activation and the Number of Apoptotic OPCs

**Fig. 7:**
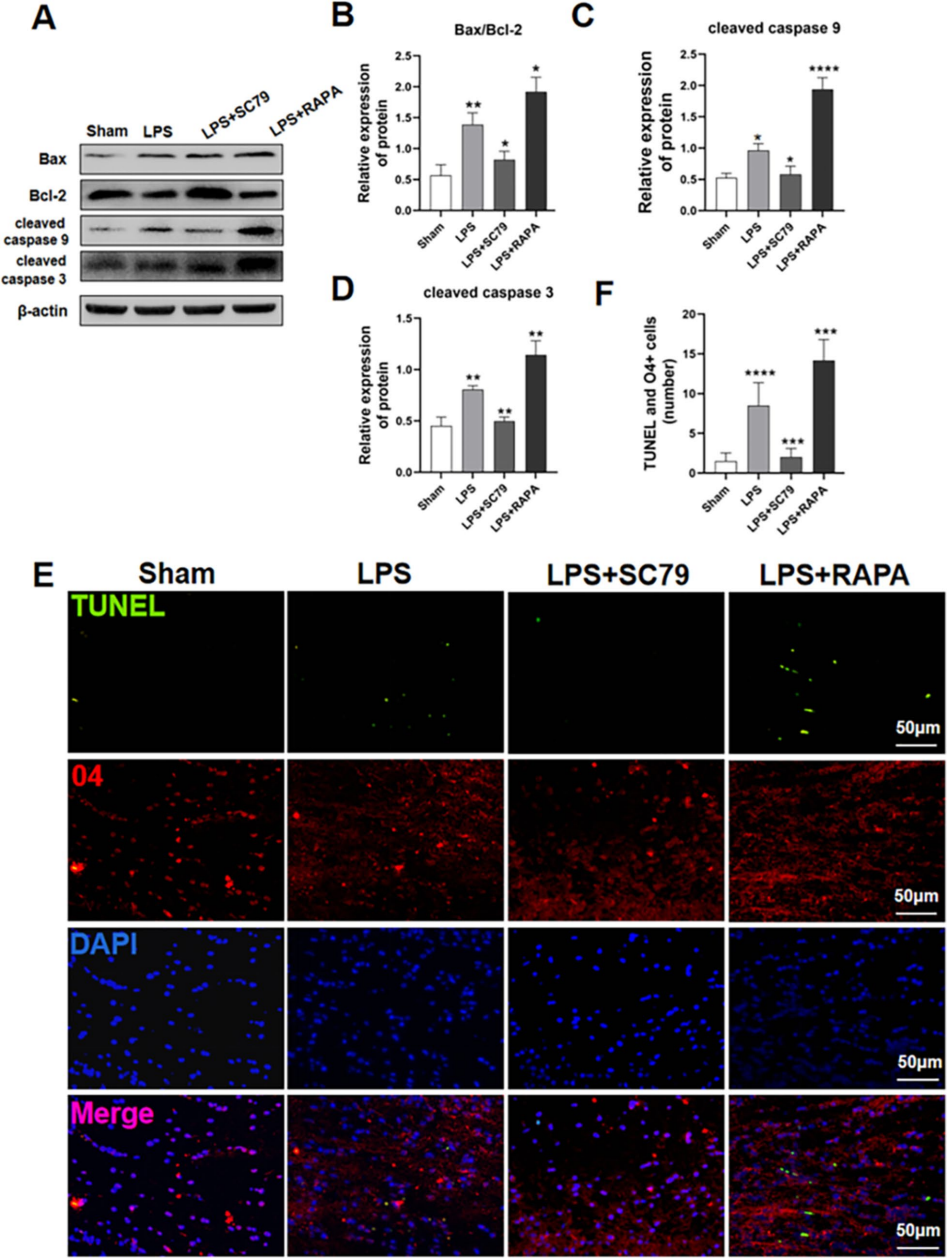
Effect of SC79 treatment on OPC apoptosis. **a**–**d** Western blot analysis of apoptotic protein caspase-9, caspase-3, and Bax/Bcl-2 expression in neonatal rat brain tissue for the Sham, LPS, LPS+SC79, and LPS+RAPA groups (Compared with the grey value of β-actin ) (∗∗P < 0.01). **e**, **f** Double fluorescence staining and quantification with anti-O4 antibody (red) and TUNEL (green). Double-positive O4+ and TUNEL+ cells were considered apoptotic OPCs (∗∗∗P < 0.001 and ∗∗∗∗P < 0.0001). n = 6; data are represented as mean ± SEM. Scale bar = 50 μm

Western blotting revealed increased Bax/Bcl-2 (P < 0.01), cleaved caspase-9 (P < 0.05), and cleaved caspase-3 (P < 0.01) expression in neonatal rat brain tissue of the LPS group compared with those in the sham-operated group rats (Fig. 7a–d). Apoptotic proteins Bax/Bcl-2 (P < 0.05), cleaved caspase-9 (P < 0.05), and cleaved caspase-3 (P < 0.01) were significantly down-regulated in the LPS+SC79 group rat tissues, whereas the opposite was observed in the RAPA treatment group rat tissues (Bax/Bcl-2, P < 0.05; cleaved caspase-9, P < 0.0001; cleaved caspase-3, P < 0.01). The TUNEL assay and O4+ double labeling (Fig. 7e–f) revealed increased TUNEL+ and O4+ cells in the brain tissue of neonatal rats in the LPS group (P < 0.0001), whereas SC79 treatment reduced the number of TUNEL+ and O4+ cells (P < 0.001). The number of apoptotic O4+ cells in the rats of the LPS+RAPA group was significantly higher than that in the LPS group rats (P < 0.001).

### Effect of SC79 Treatment on the Transition from Autophagy to OPC Apoptosis

**Fig. 8:**
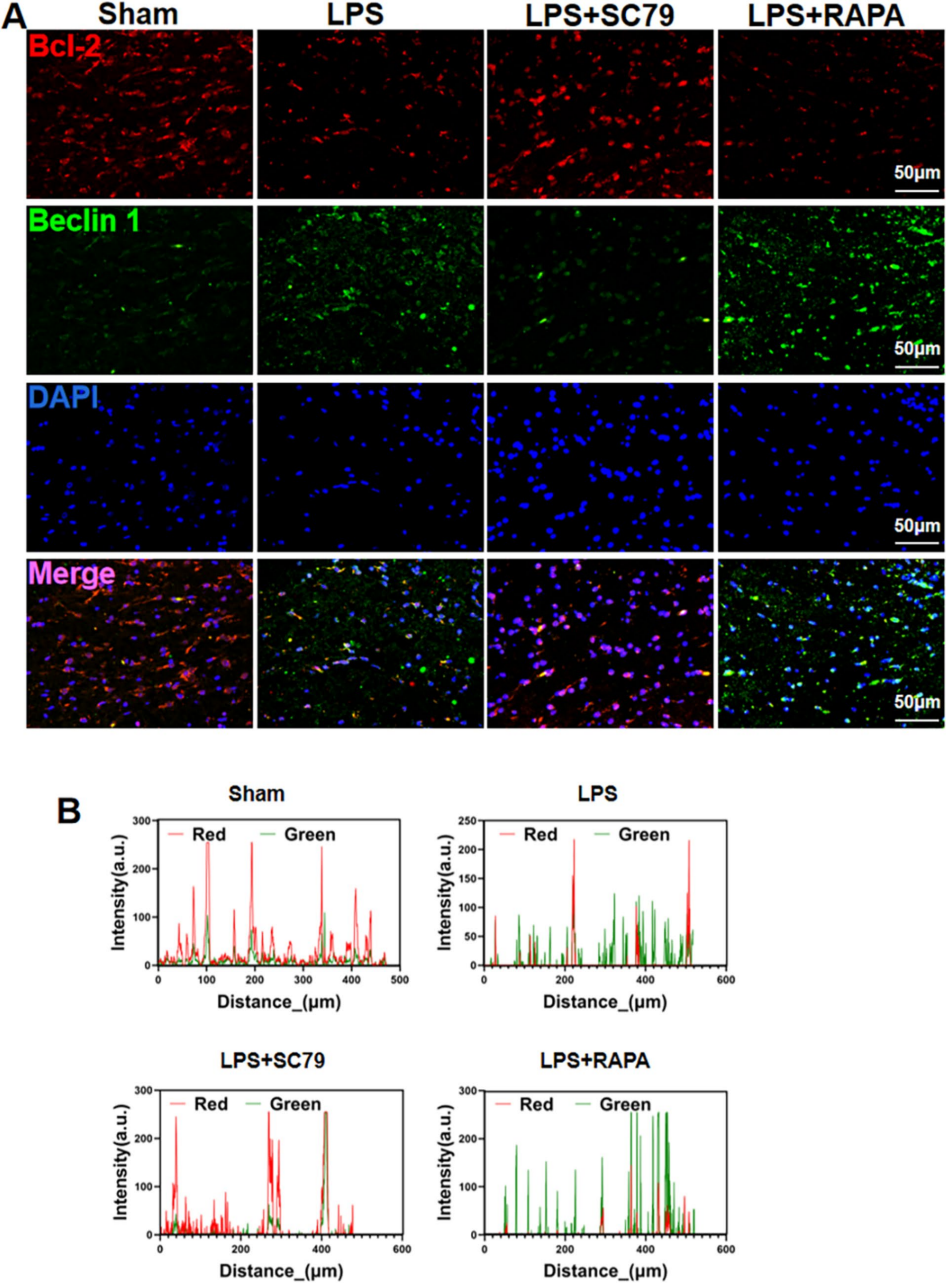
Effect of SC79 treatment on autophagy activation in white matter. **a**, **b** Immunofluorescence staining of the anti-apoptotic protein Bcl-2 (red) and autophagy initiation protein Beclin 1 (green), and their colocalization in the periventricular white matter of neonatal rat brain tissue. n = 6. Scale bar = 50 μm

Immunofluorescence and colocalization analysis showed reduced Beclin 1/Bcl-2 complexation in the white matter of neonatal rats in the LPS group compared with that in the white matter of neonatal rats in the sham-operated group (Fig. 8a, b). The LPS + SC79 group rats exhibited increased Bcl-2 expression and increased fluorescence for Beclin 1/Bcl-2 complexation, whereas LPS + RAPA group rats exhibited reduced Bcl-2 expression, increased Beclin 1 expression, and decreased Beclin 1/Bcl-2 complexation compared with that in the sham-operated group rats.

## Discussion

Various prenatal risks are associated with WMD. White matter damage is mainly caused by perinatal inflammation, such as chorioamnionitis and neonatal sepsis, hypoxic-ischemic damage associated with preterm birth, and perinatal hemodynamic instability. Gestational weeks 23–32 represent a high-risk period for WMI that coincides with a critical period of white matter development prior to myelination and a period of high susceptibility to stress damage [1]. The rodent brain on postnatal days 1–5 is equivalent to the human brain at 23–32 weeks gestation, and this time frame is therefore suitable for simulating WMI in preterm infants [21]. We used LPS to suppress OPCs development, mimicking WMD.

In this study, we found that neonatal rats in the LPS group exhibited significantly reduced body weights and slower growth rates during the vulnerable period of white matter development.However, the addition of SC79 can aid in reversing this decline. H&E staining revealed that neonatal rats in the LPS group had increased cavitation around the white matter, loose and disordered nerve fibers, and white matter damage. The intensity and depth of fluorescence in specific staining of mature oligodendrocytes using MBP + immunofluorescence staining and CNPase + immunohistochemical staining represent myelin sheath density and maturity [25]. The observed decrease in mean MBP + immunofluorescence intensity and CNPase + optical density of white matter of the LPS group therefore indicate that LPS inhibits OPC maturation and myelination.

The mechanism by which intrauterine inflammation leads to white matter maturation disorders is complex, and abnormal OPC apoptosis is considered one of the main causes of WMI [8, 23, 24, 26]. Our group previously demonstrated that in the early stages of WMI in neonatal rats, induced by intrauterine inflammation, significant up-regulation of Akt phosphorylation can reduce the number of apoptotic OPCs and protect against white matter damage [27]. However, the mechanism by which Akt protects OPCs from excessive apoptosis is unclear. Apoptosis is mediated by apoptosis proteins, including the initiating caspase, caspase-9, which is involved in the initiation of apoptosis; the effector caspase, caspase-3, which mediates apoptosis following cleavage and activation; and Bax, an important pro-apoptotic member of the Bcl-2 family, which mediates apoptosis in response to death signals. The ratio of Bax/Bcl-2 is often used to evaluate the degree of apoptosis [28]. Our results indicated that, compared with sham-operated rats, neonatal rats in the LPS group exhibited significantly increased cleaved caspase-3, cleaved caspase-9, and Bax/Bcl-2 ratios at P2. Furthermore, TUNEL+ and O4+ immunofluorescence colocalization experiments revealed significantly increased numbers of late apoptotic OPCs in the LPS group. This suggests that OPC apoptosis is an important mechanism in the pathogenesis of WMD.

Autophagy plays an important role in cell survival and death and serves as a quality control mechanism in metabolic and innate immune processes [29]. The dual role of autophagy in inflammatory diseases has recently attracted considerable attention. In several studies of acute inflammatory diseases, excessive autophagy has been shown to promote disease development [11, 12]. Beclin 1, a key initiator of autophagy, can also interact with the anti-apoptotic protein Bcl-2 to affect apoptosis. Stable Beclin 1/Bcl-2 complexation inhibits autophagy and apoptosis, whereas dissociation of Bcl-2 from Beclin 1 promotes autophagy and apoptosis [15, 30]. Autophagic flux determines the role of autophagy in WMD, playing either a protective or destructive role in different situations. Increased autophagosome accumulation, usually manifesting as increased LC3-II expression, may result from increased autophagosome formation or decreased autophagosome degradation. The linker protein p62 promotes cargo ubiquitination and can be degraded by autophagosomes as part of autophagy [31]. In this study, the autophagy marker protein Beclin 1, LC3-II and LC3-II/I ratio increased and autophagy flux protein p62 expression was significantly down-regulated in the LPS group rats compared to those in the sham group rats. Furthermore, decreased Beclin 1/Bcl-2 complexation was observed in the white matter regions of neonatal rats in the LPS and O4+ OPC groups. This indicates that abnormal OPC autophagy is up-regulated and the Beclin 1/Bcl-2 complex is degraded, thus promoting OPC apoptosis.

Multiple studies targeting inflammatory neurological diseases have demonstrated that autophagy mediated by the Akt/mTOR pathway plays an important role in this process [17, 19]. Akt directly phosphorylates and activates mTOR. As a downstream effector of Akt, mTOR negatively regulates autophagy and is associated with various downstream signaling pathways, including apoptosis and inflammatory responses [17, 18]. Zhang et al. [32] found that bicalutamide, an Akt inhibitor, and RAPA, an mTOR inhibitor, can simultaneously increase the expression of autophagy- and apoptosis-related proteins in a mouse model of cerebral ischemia. In this study, LPS injection into the lateral ventricle of neonatal rats synchronously blocked phosphorylation of Akt/mTOR pathway-related proteins, increased Beclin 1, LC3-II and LC3-II/I expression, down-regulated p62 expression, and increased apoptosis-related protein expression. Treatment with SC79 decreased Beclin 1, LC3-II and LC3-II/I expression, increased p62 expression, and down-regulated the expression of apoptosis-related proteins. RAPA treatment induced the opposite effect. These results suggest that the Akt/mTOR pathway is involved in the regulation of excessive autophagy and abnormal apoptosis in myelinating cells during WMD pathogenesis.

SC79 is an Akt phosphorylation activator with neuroprotective effects. In a study simulating cerebral ischemia and perfusion injury, Kalpage et al. [33] found that SC79 treatment maintained cytochrome serine-47 phosphorylation, reduced the level of cell death, and protected against brain injury. Miao et al. [34] found that SC79 treatment rescued propofol-induced inhibition of neural stem cell proliferation and differentiation and partially reversed propofol-induced neurocognitive impairment in a neonatal rat model. In this study, SC79 treatment increased p-Akt and p-mTOR levels and inhibited autophagy- and apoptosis-related protein expression. Yu, etc. [35] found that inhibiting mTOR phosphorylation also cut the expression of Bcl-2 protein in animal colon cancer model experiments in vivo and in vitro. Chen et al. [36] found ARNTL (a cancer gene) may adversely affect the Akt/mTOR pathway in the tongue squamous cell carcinoma model, thereby enhancing autophagy induction, as well as the expression of Bcl-2 drops. Hou, etc. ’s study [37] showed that mTOR phosphorylation was reduced, along with Bcl-2 expression in the animal model of ischemic stroke. After the resveratrol treatment, p-mTOR and Bcl-2 expression increased synchronously, neuronal apoptosis was reduced at the same time, saving brain damage.We also found that SC79 treatment also increased the binding of autophagy initiation protein Beclin 1 and anti-apoptotic protein Bcl-2, inhibited the transition from autophagy to apoptosis, and increased OPC survival, thereby promoting myelin sheath development and maturation and protecting against WMD. We speculate that SC79 inhibits Beclin 1 expression by phosphorylating Akt and subsequently phosphorylating downstream mTOR, inhibiting Beclin 1 binding to Bcl-2 and increasing Bcl-2 expression. In contrast, the RAPA treatment group exhibited decreased Akt and mTOR phosphorylation, up-regulated apoptosis- and autophagy-related proteins, reduced Beclin 1/Bcl-2 binding, increased transition from autophagy to apoptosis, increased OPC apoptosis, and further white matter damage. Promoting Akt and mTOR phosphorylation, and thus inhibiting excessive autophagy activation, may therefore reduce OPC apoptosis.

In conclusion, we determined that OPC apoptosis is an important mechanism in the pathogenesis of WMD and excessive OPC autophagy may be the cause for abnormal OPC apoptosis. SC79 treatment inhibits excessive OPC autophagy, promotes normal myelin sheath development and maturation, and reverses white matter damage by regulating the Akt/mTOR signaling pathway. These findings present novel insights into preventing WMD.

## Data Availability

Datasets are available from the corresponding author upon reasonable request.

## Abbreviations

WMD: White matter dysplasia
OPCs: Oligodendrocyte precursor cells
